# Single-Nucleotide Polymorphisms Associated with Mercury Levels and Neurological Symptoms: An Overview

**DOI:** 10.3390/toxics12030226

**Published:** 2024-03-20

**Authors:** Jamila Alessandra Perini, Jessica Vilarinho Cardoso, Alana de Oliveira Knesse, Felipe Oliveira Pessoa-Silva, Ana Claudia Santiago de Vasconcellos, Daniel Escorsim Machado, Paulo Cesar Basta

**Affiliations:** 1 Research Laboratory of Pharmaceutical Sciences (LAPESF), State University of Rio de Janeiro (UERJ), Rio de Janeiro 23070-200, Brazil; jessica_vilarinho@yahoo.com.br (J.V.C.); alanaknesse@gmail.com (A.d.O.K.); felipefacfarmacia@gmail.com (F.O.P.-S.); danielescorsim@yahoo.com.br (D.E.M.); 2 Laboratório de Educação Profissional em Vigilância em Saúde, Escola Politécnica de Saúde Joaquim Venâncio, Fundação Oswaldo Cruz (EPSJV/Fiocruz), Av. Brasil, 4365-Manguinhos, Rio de Janeiro 21040-900, Brazil; anacsvasconcellos@gmail.com; 3 Program of Post-Graduation in Public Health and Environment, National School of Public Health (ENSP), Oswald Cruz Foundation (Fiocruz), Rio de Janeiro 21041-210, Brazil; paulobasta@gmail.com; 4 Department of Endemic Diseases Samuel Pessoa, ENSP, Fiocruz, Rio de Janeiro 21041-210, Brazil

**Keywords:** mercury exposure, mercury poisoning, genetic polymorphism, toxicokinetic, neurotoxicity, environmental health

## Abstract

Mercury (Hg) pollution is a global public health concern because of its adverse effects on the environment and health. Single-nucleotide polymorphisms (SNPs) have been associated with Hg levels and outcomes. The aim of this review was to describe the research and discuss the evidence on the genetic susceptibility of Hg-exposed individuals to the development of neurocognitive disorders. A systematic review was performed to identify the genes/SNPs associated with Hg toxicokinetics and that, therefore, affect neurological function in exposed populations. Observational and experimental studies were identified by screening three databases. Thirteen articles were included (quality score 82–100%) and 8124 individuals were evaluated. Hg exposure was mainly fish consumption (77%) and, in 31% of the studies, the Hg levels exceeded the reference limits. Genetic susceptibility to higher Hg levels and neurotoxicity risk in Hg poisoning were associated with eight (*ALAD* rs1800435, *CYP3A4* rs2740574, *CYP3A5* rs776746, *CYP3A7* rs2257401, *GSTP1* rs1695, *MT1A* rs8052394, *MT1M* rs2270836, and *MT4* rs11643815) and three (*MT1A* rs8052394, *MT1M* rs2270837, and *MT2A* rs10636) SNPs, respectively, and rs8052394 was associated with both outcomes. The *MT1A* rs8052394 SNP may be used as a susceptibility biomarker to identify individuals at greater risk for higher Hg levels and the development of neurocognitive disorders in metal-exposed populations.

## 1. Introduction

**Figure 1:**
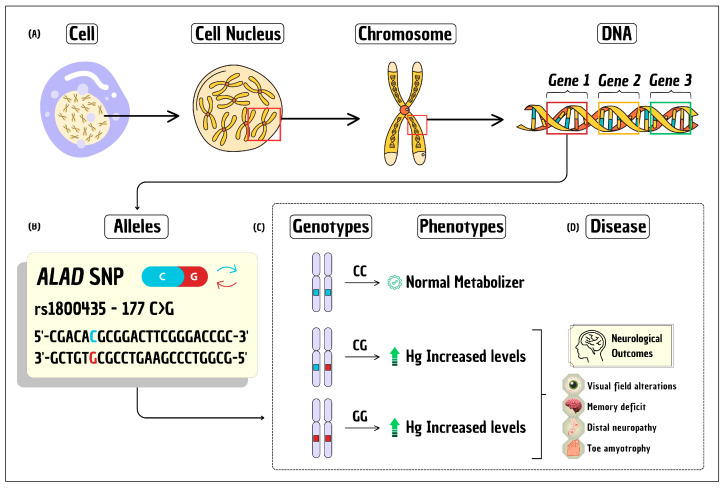
The mechanistic model to explain the basic concepts of genetic inheritance and single-nucleotide polymorphism. (**A**) Organization of human genome information in the cell. (**B**) Example of the rs1800435 SNP in the *ALAD* gene. (**C**) Example of three different genotypes of the *ALAD* rs1800435 SNP (CC, CG, and GG) and their corresponding phenotypes (normal and poor metabolizers). (**D**) Example of neurological outcomes caused by carrying the *ALAD* rs1800435 SNP. Legend: SNP = single-nucleotide polymorphism.

The human genome consists of approximately 3 billion base pairs, which are organized into 46 nuclear chromosomes that carry the genomic information from cell to cell. Human genes (~20,000 protein-coding genes) are the basic unit of inheritance, encoding information that determines physical and biological traits. Genes are organized into chromosomes in each nucleated cell (Figure 1A). Specific base pair sequences (A-T and G-C) that carry genetic information and their variation between individuals is minimal, only 0.1% of the human DNA sequence. This variability among individuals is due to the presence of different alleles for the same gene (Figure 1B), resulting in different genotypes (Figure 1C), which may or may not differ in phenotypes (e.g., eye color or disease such as sickle cell anemia) (Figure 1D). The genotype, a combination of two alleles, is inherited from ancestors, with one coming from each parent [1].

Regarding the genetic basis of a diseases, there are two main variations in genes: (i) the mutated allele, which is associated with the development of the pathological condition, and it is observed in the human population at a frequency of less than 1%; and (ii) the allelic variant (polymorphism), which may or may not be associated with a greater susceptibility to develop a pathology compared to other individuals. However, there are neutral mutations, those that provide no significant advantage or disadvantage to the individual, they exist because of the inherent randomness of the mutation process. Polymorphisms are the most common genetic variations (≥1%) and most do not have serious consequences for human health. A genomic variation at a single base position in the DNA is called a single-nucleotide polymorphism (SNP) [2] (Figure 1B). It is important to highlight that lifestyle and environmental factors also play a critical role in disease susceptibility; and the SNPs that affect susceptibility to a particular phenotype, such as slow metabolizers of xenobiotics, may have originally been selected for other reasons unrelated to exposure to the toxic agent [3]. 

Recently, SNPs in the human genome have been extensively investigated as potential biomarkers of disease and drug response to understand the usefulness of these genetic variations in human health and other traits. In regard to global environmental pollutants, the use of SNPs in genetic testing may help to identify individuals at risk of developing a disease early and/or having higher levels of these agents in the body [3,4,5,6]. Among environmental pollutants, mercury (Hg) is one of the most toxic on the planet and poses a significant threat to human health. It can be detected in any location on the planet and in distinct environmental compartments (i.e., soil, sediment, rivers, lakes, oceans, etc.). Although there are natural sources of Hg on the planet, such as erupting volcanoes and the thawing of permafrost, anthropogenic activities are more relevant sources because they cause the release of large quantities of mercury into nature. In 2013, the Minamata Convention on Mercury was established to banish the use of Hg in all industrial processes and to control and replace the use of Hg in artisanal gold mining [7]. Currently, gold mining can be considered the major anthropogenic source of Hg to the environment [4], but other sources such as thermoelectric power plants, cement factories, and fossil fuel combustion also contribute to the emission of tons of atmospheric Hg. Such activities greatly alter the biogeochemical cycling of Hg, increasing the risk of human exposure and subsequent disease [7].

Human Hg exposure could occur via different routes. In the general population, the most common exposure routes are the ingestion of methylmercury (MeHg) contaminated fish and the use of dental amalgam with metallic mercury (Hg^0^). There are also cases of occupational exposure, which mainly involve mining workers and dentists. After ingestion or inhalation (i.e., methylmercury and metallic mercury, respectively), Hg is rapidly absorbed, distributed to the tissues, and slowly eliminated, and Hg exposure has been associated with neurocognitive disorders and other health outcomes [4,5,8]. The overall scenario presented makes it clear that Hg contamination of the environment is a public health problem of global importance [7]. 

The SNPs in genes involved in Hg toxicokinetics may be associated with severe Hg poisoning [4,5,6,9] and it may help to identify susceptible subgroups at risk in exposed populations. Therefore, a systematic review was performed to: (i) introduce basic concepts on genetic polymorphisms; (ii) identify the genes/SNPs associated with Hg toxicokinetics; (iii) describe the frequency of these SNPs among ethnic groups; (iv) discuss the pathways through which Hg toxicity and genetic susceptibility may affect neurological function in exposed populations; and (v) discuss the different Hg exposure scenarios and the use of exposure biomarkers. This review can help to expand and strengthen the capacity for identification, diagnosis, treatment, and monitoring of Hg-exposed populations. 

## 2. Materials and Methods

### 2.1. Information Source and Search Strategies

A bibliographical search of articles published in the last ten years (2013 to 2023) was performed to identify relevant studies addressing the SNPs associated with toxicokinetics and neurocognitive disorders of Hg poising. This time interval was chosen due to the advancement of bioinformatics tools for identifying SNPs and the establishment of different methodologies for genotyping these genetic markers over the past decade. Three electronic databases (PubMed, Medline, and Scielo) were searched using the following combined descriptors in English: (“metabolic enzymes” or “toxicokinetic”) and (“polymorphism” or “SNP” or “genetic polymorphism”) and (“methylmercury” or “mercury”). 

### 2.2. Eligibility Criteria

A systematic review was conducted to identify the genes/SNPs associated with Hg toxicokinetics and to discuss the pathways by which Hg toxicity and genetic susceptibility may affect neurological function in exposed populations. The articles selected for this review followed the following inclusion criteria: (i) observational studies (cross-sectional, cohort, case series, case studies, and reports) or experimental studies related to the topic of the present systematic review; (ii) SNPs linked to Hg toxicokinetics; (iii) studies with any information on neurological symptoms associated with Hg levels; and (iv) articles that evaluated human hair, blood, or urine samples. The exclusion criteria were: (i) publications that did not analyze SNPs; (ii) did not include quantification of mercury exposure; (iii) analysis of Hg in matrices other than hair, blood, or urine samples; (vi) animal or in vitro research; (v) reviews and meta-analysis; (vi) research published in languages other than English; and (vii) publications without the full text accessible. 

### 2.3. Data Extraction

The following information was extracted from each article after the studies were selected according to the eligibility criteria: first author; year of publication; study population; type of exposure source; number of participants; age; sex; body mass index (BMI); Hg dosage method; details of the SNPs/genes studied; genotyping technique to identify SNPs; allele frequency data; Hg levels; and data on the association of Hg levels with SNPs and neurological symptoms after adjustment and correction tests. Additional details of genes and SNPs were searched on the NCBI [10] and/or SNPedia [11] platforms.

### 2.4. Quality Assessment of Studies

All of the included studies were independently analyzed by three reviewers (JVC, AOK, and FOPS) using the STROBE checklist used for observational studies and the CONSORT checklist used for randomized clinical trials. STROBE has twenty-two topics related to the title and abstract, introduction, methodology, results, discussion, and financing of each study, generating a score of 0 or 1 for each item. CONSORT consists of a 25-item checklist, which focuses on reporting the design, analysis, and interpretation of the trial, also generating a score of 0 or 1 for each item. Publications with a score greater than 80% are considered good-quality studies [12,13].

### 2.5. Analysis and Illustrations

The chi-square test was performed to verify the difference in the allelic frequencies of the studied SNPs between the articles included in this systematic review. 

The figures of the mechanistic model to explain the basic concepts of genetic polymorphisms and the hypothesis of SNPs associated with genetic susceptibility to neurotoxicity in Hg-poisoned populations were developed using Canva [14].

## 3. Results

**Figure 2:**
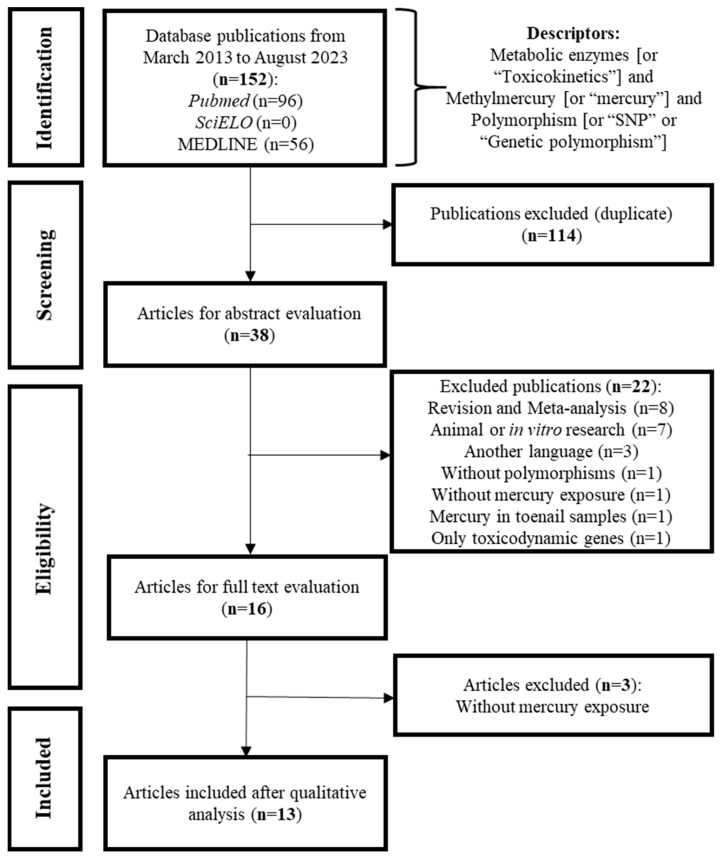
Flowchart of the literature search, screening, eligibility, exclusion, and inclusion of articles evaluated in this review.

Using the proposed search strategy, 152 articles were found in 3 databases and, after the exclusion and the full text evaluation, 13 studies [5,15,16,17,18,19,20,21,22,23,24,25,26] were included in this review (Figure 2). 

The quality score ranged from 82 to 100%, and 84.6% showed a percentage of study quality greater than 90% according to the STROBE and CONSORT checklists. Table 1 describes the basic information on the population studied by continent (South and North America, Europe, Africa, and Asia), and one publication studied more than one population: European and African [22]. Thus, 8124 individuals were included in this review, 58.8% were males, and ages ranged from 0 to 87 years (Table 1). Four articles studied only children [5,16,22,25], eight only adults [15,17,18,19,20,21,23,24,26] and one both children and adults [23] (Table 2). 

Table 2 shows the different mercury exposure routes, the population groups studied, the mercury levels in exposure biomarkers, and indicates the studies which the mercury concentration in biological matrices exceeds the reference limits, among the articles obtained in this review systematic. Three mercury exposure routes were identified: fish consumption, use of dental amalgams and occupational exposure (dental office’s professionals). The consumption of methylmercury contaminated fish was indicated as a priority exposure route in all identified studies, except in one article, which the priority route is the use of dental amalgams [16]. Only two articles considered a double route of mercury human exposure: occupational plus fish consumption [20] and dental amalgamation plus fish consumption [26]. Three distinct population groups were identified: urban (61.5%), indigenous (23%) and riverside (15.4%). The exposure biomarkers used were blood (38.4%), hair (30.7%), urine (7.7%), umbilical cord blood + hair (7.7%), blood plus hair (7.7%), blood plus hair plus urine (7.7%). To interpret the mercury dosage in biomarkers, different parameters recommended by international health agencies were used. For hair samples, the limit of 6.0 μg/g, recommended by the FAO/WHO (1989) [27], was used. For the blood samples, including umbilical cord blood, we used the limit of 8.0 μg/L recommended by the Canadian government [28] and for the urine samples we used the limit of 10 μg/L recommended by the WHO (2008) [29]. In four articles (31%), the mercury levels exceeded the proposed exposure limits [5,15,18,22].

The SNP information of the genes studied in individual exposure to Hg are described in Table 3 and Table S1 by the gene name. In order to identify SNPs with a relatively high frequency (above 10%), which is one of the reasons justifying the identification of this SNP as a genetic biomarker to evaluate individuals at risk, the minor allele frequency (MAF) of the SNPs was also included in Table 3 and Table S1. A total of 113 SNPs in 74 genes were analyzed, and the SNPs localization in the genome were: 10.6% 5′UTR, 11.5% promotor, 46.0% exon, 15.9% intron, 15.1% 3′UTR, and 0.9% intergenic. In addition to the toxicokinetic genes, the included articles also investigated 13 SNPs in 11 different toxicodynamic genes: *BCL11A*, *BDNF*, *CAT*, *GLRX2*, *HMOX1*, *NOS1*, *PRDX2*, *PRDX6*, *TXNRD2*, *TXNRD3*, and *VDR*. TaqMan real-time PCR (69.2%) was the main method used for genotyping analysis. In addition, two articles used TaqMan and another technology [17,22]; two articles from the same group used MassArray technology [20,24], another used a spectrophotometer [21], and one used RFLP-PCR [26] (Table S1). According to Dresher and colleagues (2014) [17], the genotyping error rate of MassArray technology (~0.5%) was higher than TaqMan PCR (~0.1%). 

The MAF (Table 3 and Table S1) of all investigated SNPs ranged from 0.6% (*MT4* rs11643815) in the Chinese population [23] to 92% (*CYP3A7* rs2257401) in the Italian population [22]. However, the frequencies of the same SNPs were significantly different (*p* < 0.001) to compared to the USA population (*MT4* rs11643815, 9%) [20], and the African population (*CYP3A7* rs2257401, 45–47%) [22]. Sixteen (14.2%) SNPs were investigated in ≥2 articles and three SNPs were studied in ≥3 articles: *GCLC* rs17883901 SNP with similar frequencies in Brazilian (MAF not informed: ~9%) [18], US (8%) [20], and Chinese (8%) [23] populations; *GSTP1* rs1695 with significantly different MAF (*p* < 0.001) in African (47%) [25], Brazilian (43%) [15,18], US (33%) [20], and Chinese (18%) [23] populations; and *MT2A* rs10636 with similar frequencies in the US population (25%) [20] and the European Portuguese (23–25%) [16] and Chinese populations (22%) [23]. In addition, *CYP3A4* rs2740574, *CYP3A5* rs776746, and *CYP3A7* rs2257401 have been studied in three European countries (Spain, Italy, and Greece) and African populations with significantly different MAF (Table 3) between European and African [22]: *CYP3A4* rs2740574 (1–4% vs. 54%, respectively), *CYP3A5* rs776746 (6–8% vs. 55%, respectively) and *CYP3A7* rs2257401 (88–92% vs. 46%, respectively) (*p* < 0.001). For the *CYP3A7* rs2257401 SNP, there was also a significant difference between the Italian and Greek populations (92% and 88%, *p* = 0.03). Noteworthy are the significant differences (*p* < 0.001) in MAF of other SNPs studied in distinct populations (Table 3 and Table S1): *GPx1* rs1050450 with 3% in Chinese [23] and 25% in Brazilian [19]; *MT1M* rs2270836 with 22% in Chinese [23] and 35% in the US population [20]; and six more SNPs were significantly different (*p* < 0.001) between the North American (US) [20] and Canadian populations [24], respectively: *ATP7B* rs1061472 (27% and 48%) and *rs1801243* (27% and 48%), *CAT* rs7943316 (43% and 10%), *MTHFR* rs2274976 (9% and 49%), *MTRR* rs1801349 (47% and 27%), and *SLC43A2* rs4790732 (34% and 49%). Only the MAF of the *GSTA4* rs405729 SNP was similar between them (39% and 35%) [20,24]. The *GCLM* rs41303970 SNP has been studied in Brazilian (18%) [15] and Chinese (20%) [23] populations, and no significant difference was observed between the two groups. The *ALAD* rs1800435 SNP was studied in two Brazilian groups from different regions (1% southeast and 3% north) and there were no significant differences between them [5,18] (Table 3 and Table S1). 

The SNPs (10%) in genes involved in toxicokinetics that were significantly associated with Hg levels and/or neurological outcomes after adjustment and correction tests are described in Table 4 by gene name, with their biological pathways (transporters and metabolic enzymes). Overall, eight SNPs were associated with higher Hg levels [18,20,22,23,25,26]. One SNP (*GSTP1* rs1695) was associated with higher Hg levels in two different studies [23,25], twelve SNPs were associated with low Hg levels [15,17,20,23,24] and two found no significant association [16]. Regarding neurotoxicity (Table 4), only three articles (30%) performed neurological assessment and found SNPs in toxicokinetic genes associated with any neurological changes after adjustment and correction tests in individuals exposed to Hg poisoning [16,22,26]. 

**Figure 3:**
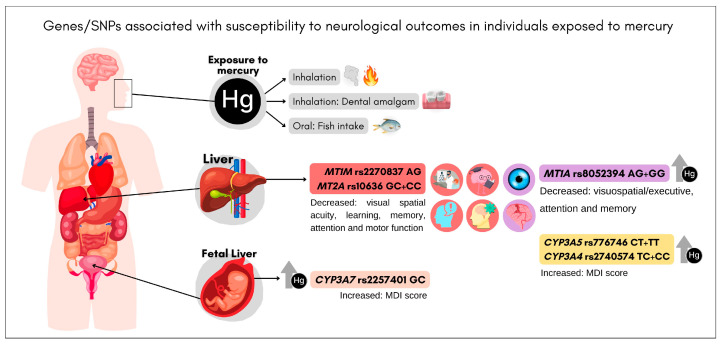
Mechanistic model to explaining the basic concepts of genes/SNPs associated with genetic susceptibility to any neurological changes in individuals exposed to mercury poisoning. Legend: MDI = mental development index. Mercury exposure routes: fish consumption, use of dental amalgams, and occupational exposure. *CYP3A* SNPs were inversely associated with increased Hg levels and increased MDI scores. The *MT1A* SNP was associated with higher Hg levels and cognitive impairment in Hg poisoning. The *MT1M* and *MT2A* SNPs were associated with negative changes for the neurobehavioral functions associated with Hg exposure among boys.

Figure 3 describes these SNPs associated with genetic susceptibility to the development of neurological symptoms in individuals exposed to Hg. SNPs in genes involved in toxicokinetics were associated with visual field changes (*MT1M* rs2270837, *MT2A* rs10636, and *MT1A* rs8052394), learning and/or memory deficits (*MT1M* rs2270837, *MT2A* rs10636, and *MT1A* rs8052394), attention deficit (*MT1M* rs2270837, *MT2A* rs10636, and *MT1A* rs8052394), and reduced motor function (*MT1M* rs2270837 and *MT2A* rs10636) [16,26]. Sirivarasai and colleagues studied 436 adults (58 ± 3 years old) from Thailand [26] and Woods and colleagues studied 507 children (8–12 years old) from dental amalgam tooth filling work in Portugal [16]. Three other SNPs were inversely associated with increased Hg levels and increased mental development index (MDI) scores (*CYP3A5* rs776746, *CYP3A4* rs2740574, and *CYP3A7* rs2257401) in 2639 children from three birth cohort studies from Africa (20–30 months of age), Spain (14 months), and Italy and Greece (18 months of age) [22].

## 4. Discussion

Genetic differences between individuals may contribute to susceptibility to Hg neurotoxicity. Gene–environment interactions have been studied to explain how genes may influence the toxicokinetic and neurological effects of Hg [3,4,5,6,8,30]. In this context, the aim of this review was to assess the current state of knowledge on SNPs on toxicokinetic genes and their interactions on Hg neurotoxicity by reviewing the literature over the last 10 years.

Eight SNPs were identified in genes encoding enzymes involved in toxicokinetics (*ALAD* rs1800435, *CYP3A4* rs2740574, *CYP3A5* rs776746, *CYP3A7* rs2257401, *GSTP1* rs1695, *MT1A* rs8052394, *MT1M* rs2270836, and *MT4* rs11643815) associated with higher Hg levels [18,20,22,23,24,25,26]. The adverse neurological outcomes were observed in Hg-poisoned individuals associated with three SNPs in genes encoding members of the metallothionein family, which bind various heavy metals: *MT1A* rs8052394, *MT1M* rs2270837, and *MT2A* rs10636 [16,26]. Furthermore, the *MT1A* rs8052394 SNP was associated with both higher Hg levels and neurotoxicity in Hg poisoning [26]. Surprisingly, three SNPs in the cytochrome P450 3A (*CYP3A*) family genes showed significantly opposite effects on Hg levels and outcomes. *CYP3A* SNPs were associated with a doubling of cord blood Hg levels, while children also had higher MDI scores [22].

### 4.1. CYP3A (rs2740574, rs776746, and rs2257401)

Cytochrome P450 (CYP) enzymes function as the primary defense against exogenous chemicals, which are indiscriminate toward substrates with lipophilicity and large molecular weight and, therefore, they play a vital role in protecting the body from toxic derivatives of harmful compounds. The CYP3A, a major subfamily, is encoded by a 231 kbp cluster (chromosome 7q21.1) that includes the genes *CYP3A4*, *CYP3A5*, and *CYP3A7* [31,32]. The allelic distribution and expression of the *CYP3A4*, *CYP3A5*, and *CYP3A7* genes varies from one population to another around the world, with strong interindividual and geographic differences [33,34,35]. *CYP3A4* rs2740574 (−392T>C) SNP in the promoter region results in lower expression of the enzyme [33]. *CYP3A5* rs776746 (6986 C>T) SNP causes defective mRNA splicing and reduced functional protein synthesis [32,35]. *CYP3A7* rs2257401 (26,041G>C, Thr409Arg) SNP promotes decreased enzyme activity [34,36]. In accordance with these genetic changes, higher Hg accumulation has been observed in individuals who carry these three variant alleles of CYP3A. Interestingly, however, in the study by Llop and colleagues [22], individuals carrying the variant alleles of CYP3A4, CYP3A5, and CYP3A7 also had higher MDI scores. A plausible biological reason for this contradictory finding is that CYPs may metabolize fish nutrients that are beneficial for brain development, such as vitamins, fatty acids, or selenium, with equal efficiency. Therefore, in addition to accumulating Hg, children also accumulate important nutrients. However, the involvement of *CYP3A* in the development of Hg neurotoxic outcomes remains unclear [22,37]. It is important to highlight that high Hg contamination is a worrisome risk factor that may trigger disease processes independent of the presence of SNPs in genes associated with Hg metabolism and excretion.

### 4.2. ALAD (rs1800435)

The enzyme delta-aminolevulinic acid dehydratase (ALAD) is essential for heme synthesis and plays an important role in metal toxicokinetics, primarily facilitating metal transport throughout the body. The *ALAD* rs1800435 (chromosome 9q34) SNP (177C>G, Lys59Asn) gives rise to an alternatively functional isozyme characterized by a distinct protein binding affinity, particularly for heavy metals [38]. Individuals carrying the variant allele *ALAD* rs1800435 G have a decrease in the activity of the enzyme, which can lead to an increase in Hg in the body and consequently to neurological disorders [5,18], such as changes in the visual field, memory deficits, distal neuropathy and amyotrophy of the toes [5].

### 4.3. GSTP1 (rs1695)

The glutathione S-transferase Pi 1 (GSTP1) is one of the members of the GST superfamily (phase II enzyme) that catalyzes the conjugation of the small tripeptide glutathione (GSH) with MeHg, promoting its elimination in bile through the ABC transporter system [39]. The *GSTP1* rs1695 (chromosome 11q13.2) SNP (313A<G, Ile105Val) leads to a decrease in catalytic efficiency and excretion of foreign substances. This SNP has been previously associated with increased Hg levels [23,25,40,41,42], neurological outcomes [6,41,43], and nephrotoxicity induced by Hg [42]. Here, two articles observed *GSTP1* rs1695 SNP as significantly influencing the accumulation of Hg in the human body [23,25], which means it stands out as candidate SNP for mapping susceptibility in individuals at risk of developing early signs of Hg poisoning.

### 4.4. MTs (rs8052394, rs2270836, rs2270837, rs10636, and rs11643815)

Another candidate SNP that may help identify the genetic susceptibility of individuals at risk for developing early signs of Hg poisoning to mitigate the widespread effects of metal poisoning is *MT1A* rs8052394 (152A>G, Lys51Arg) on chromosome 16q13. Individuals carrying the variant genotypes have either higher Hg levels [26,43] and reductions in scores on the attention and memory sub-domains [26]. The *MT1A* SNP may accelerate the development of Hg-induced neurotoxicity, leading to uncontrolled oxidative stress, cellular damage, inflammatory responses, and multiple pathological stages [26,44]. Metallothioneins (MTs) are proteins that play an important role in metal detoxification, mercury homeostasis, and antioxidant activities [45]. Thus, several SNPs in *MTs* genes were associated with altered genetic susceptibility to Hg levels in exposure from different sources [40,46,47,48,49]. Three SNPs located in the 3′UTR region of the *MT1M* (rs2270836 and rs2270837) and *MT2A* (rs10636) genes affect the stability of their respective mRNA and protein translation [50]. The *MT1M* rs2270836 SNP was associated with greater Hg accumulation in urban populations with occupational and fish consumption sources [20]. The *MT1M* rs2270837 and *MT2A* rs10636 SNPs had a significant effect on neurobehavioral test performance and Hg exposure in boys, particularly on visual spatial acuity, learning and memory domains, and perceptual cognition due to Hg exposure [16]. Similarly, the *MT4* rs11643815 (Gly48Asp) SNP, which is involved in transcriptional activity, protein structure and may result in an impaired metal-binding protein [51], was associated with higher Hg levels [20].

### 4.5. Insights and Other Features

When we analyzed the different Hg exposure scenarios presented in the articles obtained through this systematic review, we noticed a strong heterogeneity among the studies. Firstly, it is necessary to consider the different routes of exposure to Hg identified in the articles. Although fish consumption was the most cited exposure route in the studies, the amount of MeHg ingested by different population groups is a reflection of the Hg contamination degree and the frequency of fish consumption in the diet, which is in many instances influenced by cultural aspects. For example, the works developed by Barcellos et al. (2013 and 2015) [15,18] and Perini et al. (2021) [5] investigated groups living on the Tapajós River banks (Amazon, Brazil), which is seriously impacted by illegal gold mining. In these cases, the amounts of Hg detected in exposure biomarkers was up to six times the limit established as a reference (48.5 µg/L in blood samples) [15]. It is also important to highlight that riverside and indigenous populations tend to consume larger quantities of fish, which can increase the intake of environmental contaminants. Although both populations had high exposures to Hg, the neurological effects were different, demonstrating the importance of individual susceptibility due to the presence of SNPs that can lead to differences in the accumulation, distribution, and clearance of Hg [9]. The opposite situation can be observed in studies that investigated urban populations. In these cases, Hg levels in biomarkers were much lower than the limits established for hair and blood samples. Most of the time, lower Hg levels are the result of more diverse diets in which fish is not the only source of protein. The only exception is observed in a work evaluating 2639 children from different cohort studies, where Hg levels detected in cord blood samples were higher than the limit in the population group from the Seychelles and Spain [22]. However, regardless of the route of exposure, Hg can cross the blood–brain barrier and cause neurological changes. The goal of studying SNPs in genes involved in the Hg toxicokinetic pathway is to identify any genetic variation that may influence the accumulation of the metal in the body and, consequently, early neurotoxic effects. The three SNPs associated with Hg neurotoxicity (*MT1A* rs8052394, *MT1M* rs2270837, and *MT2A* rs10636) are located in pathway genes of metallothioneins, which play an important role in metal detoxification [16,26,46]. Among these SNPs the *MT1A* rs8052394 SNP was associated with neurotoxicity and higher Hg levels [26]. It is worth noting that high-Hg contamination is an important environmental risk factor, which can trigger illness processes in the local population, regardless of the presence of genetic polymorphisms that make Hg excretion difficult.

Age is also an important factor as there is a correlation between cumulative Hg exposure and neurological outcomes. The molecular and cellular changes in the brain caused by mercury are closely linked to neurological disorders such as memory loss, learning difficulties and motor dysfunction. The characteristics and mechanisms of MeHg poisoning differ between adults and children, with fetuses showing increased susceptibility to MeHg compared to adults, as evidenced by exposure events that have shown that acute or chronic MeHg exposure can lead to adverse effects at all stages of development [52]. Two published studies followed 1022 newborns exposed to prenatal MeHg and assessed neurobehavioural effects at 7 and 14 years of age. There was a significant association of this exposure with motor, visual, attention, memory, and verbal deficits, suggesting long-lasting neurotoxic effects on central nervous system function [53,54]. The occurrence and severity of these neurological effects depend on the dose and time of exposure, the route of entry into the body, age, and individual genetic susceptibility. Genetic susceptibility has also been associated with visual field changes, memory impairment, distal neuropathy, and toe amyotrophy in children with Hg levels above the reference limit [5].

In addition, Hg exposure as an environmental concern is relatively recent, and existing genetic polymorphisms may not have undergone the evolutionary pressure of natural selection to cope with significant Hg exposure. Genetic evolution is a dynamic process that constantly adapts to changing environments. The adverse effects of Hg on human health are increasingly recognized as a global public health problem. However, the mechanisms of action, as well as the genetic aspects of susceptibility to Hg toxicity, are not fully understood. 

In the current review, three SNPs (*MT1A* rs8052394, *MT1M* rs2270837, and *MT2A* rs10636) were associated with neurotoxicity. The impact of these SNPs on neurological outcomes in Hg-exposed individuals requires replication in other groups for early identification and protection of susceptible individuals from Hg toxicity. Statistical correlation studies are also needed to understand the true relationship between the SNPs, the Hg levels, and the outcomes. With the advent of high technology, the possibility of mass genotyping at low cost is rapidly becoming a reality. However, an individual’s genetic information must be used responsibly and ethically in the context of public health. Individuals must fully understand the risks and benefits of genetic testing and have the autonomy to decide whether or not to undergo this diagnosis. Equity in access to health services and preventive interventions is necessary. In addition, individuals identified as being genetically predisposed to negative outcomes may face community stigma, a lower likelihood of receiving job offers, poor access to health services, psychosocial problems, and others. It is necessary to fully understand the exposure, mechanisms of action, and potential risks of Hg and genetic susceptibility in order to develop public health strategies, a comprehensive action plan, public health initiatives, and other preventive approaches to reduce health effects in exposed populations, especially vulnerable groups.

## 5. Conclusions

The current study provides evidence for candidate SNPs (*ALAD* rs1800435, *CYP3A4* rs2740574, *CYP3A5* rs776746, *CYP3A7* rs2257401, *GSTP1* rs1695, *MT1A* rs8052394, *MT4* rs11643815, *MT2A* rs10636, and *MT1M* rs2270836 and rs2270837) involved in individual genetic susceptibility to show higher Hg levels and neurological outcomes. *MT1A* rs8052394 is the most important SNP that can be incorporated into clinical practice as a susceptibility biomarker to identify individuals at higher risk of presenting with higher Hg levels along with early neurological changes in vulnerable populations exposed to metals. These results corroborate previous findings describing genetic susceptibility to Hg neurotoxicity in exposed populations and may help to formulate public health strategies for future health interventions aimed at protecting at-risk individuals.

## Figures and Tables

**Table 1:** Main characteristics and quality of publications included in review (n = 13).

Reference	Population	Number	Sex (Male)	Age (Years)	BMI (kg/m^2^)	Quality Assessment (%) ^a^
[15]	South America (Brazil)	400	51.7	41.8 ± 16	25.7 ± 4.2	91
[18]	South America (Brazil)	395	52.4	40.5 (18–87)	24.0	91
[19]	South America (Brazil)	149	0	26.6 ± 7.8 (18–48)	22.2 ± 2.6 (17–30)	91
[5]	South America (Brazil)	103	39.8	6.6 ± 4.5 (0–14)	16.0± 1.3 (14–22) ^b^ 18.8 ± 1.9 (15–22) ^c^ 0.2 ± 0.8 (−1.7–1.8) ^d^	95
[21]	South America (Brazil)	959	43.9	54.7 ± 10.4	28.0 ± 5.2	82
[17]	North America (Canada)	881	41.9	40.1	33.8	100
[24]	North America (Canada)	665	100	37.2 ± 14.1 (18–76)	27.4 ± 5.7 (17–47)	95
[20]	North America (USA)	380	62.6	54.8 ± 11.4 (25–82)	-	95
[22]	Europe (Spain)	625	52.6	1.1 ± 1.3	-	95
[22]	Europe (Italy)	573	51.6	1.6 ± 0.9	-	95
[22]	Europe (Greece)	281	44.7	1.5 ± 1.1	-	95
[16]	Europe (Portugal)	507	49.7	10.2 ± 0.8 (8–12)	-	100 ^e^
[22]	Africa (Seychelles)	211	48.8	2.3 ± 1.2	-	95
[22]	Africa (Seychelles)	949	50.8	1.7 ± 1.3		95
[25]	Africa (Jamaica)	266	81.6	(2–8) ^f^	-	95
[23]	Asia (China—Children)	179	54.3	4.6 (4–5)	14.9 (14–16)	95
	Asia (China—Mothers)	165	0	36.0 (33–40)	22.0 (20–25)	95
[26]	Asia (Thailand)	436	65.1	58.8 ± 3.0	24.8 ± 3.7	86

BMI = body mass index (kg/m^2^). Age and BMI are mostly shown as means ± standard deviation (range). (-) no information. ^a^ Score according to the STROBE checklist used for observational studies. ^b^ Children aged between 6 and 10 years; ^c^ Children aged over 10 years; ^d^ Z-score for children aged between 0 and 5 years; ^e^ Only in the Wood et al., 2013 [16] article was the CONSORT checklist used. ^f^ Around 30% of children were over 3 years old.

**Table 2:** Analysis of the mercury content and levels in three different biological matrices (n = 13).

Reference	Hg-Exposure Route	Population Group	Cord Blood (µg/L)	Blood (µg/L)	Hair (μg/g)	Urinary (μg/L)	≥Hg-Reference Limit (Yes or No) ^f^
[15]	Fish consumption	Riverine—Adults	-	48.5 ± 36.5	13.8 ± 10.2	-	Yes
[17]	Fish consumption	Indigenous—Adults	-	3.3 (3.0–3.7)	-	-	No
[18]	Fish consumption	Riverine—Adults	-	39.8	-	-	Yes
[20]	Occupational and fish consumption	Urban—Adults	-	3.7 ± 3.9	0.6 ± 1.0	1.3 ± 1.8	No
[19]	Fish consumption	Urban—Adults	-	-	0.6 ± 0.7 (0.0–4.4)	-	No
[21]	Fish consumption	Urban—Adults	-	1.4 (1.3–1.5)	-	-	No
[23]	Fish consumption	Urban—Adults	-	-	0.9 (0.7–1.4) ^b^	-	No
[24]	Fish consumption	Indigenous—Adults	-	-	1.0 (0.7–1.4)	-	No
[26]	Dental amalgam and fish consumption	Urban—Adults	-	6.3 (0.8–27.6)	-	-	No
[16]	Dental amalgam	Urban—Children	-	-	-	1.3 ± 3.0 ^c^ 1.8 ± 2.3 ^d^ (0.0–31.7) ^e^	No
[22]	Fish consumption	Urban—Children	39.3 ± 25 (Seychelles)	-	5.8 (Seychelles)	-	Yes
			11.3 ± 9 (Spain)	-	-	-	Yes
			7.5 ± 5 (Greece)	-	-	-	No
			5.6 ± 4 (Italy)	-	-	-	No
[23]	Fish consumption	Urban—Children	-	-	1.0 (0.7–1.5) ^a^	-	No
[25]	Fish consumption	Urban—Children	-	1.0 ± 1.3	-	-	No
[5]	Fish consumption	Indigenous—Children	-	-	7.0 ± 4.5 (1.4–23.9)	-	Yes

(-) no information.; ^a^ child population; ^b^ mother population; ^c^ boys; ^d^ girls; ^e^ the measurement unit used was μg of Hg per g of creatinine. ^f^ Hg reference limit for each biological matrix: hair ≥ 6.0 μg/g, blood and cord blood ≥ 8.0 μg/L, and urine ≥ 10 μg/L.

**Table 3:** Details of genes and SNPs associated with mercury levels and/or neurological outcomes.

Gene	Chr	dnSNP ID	Gene Locus	SNP Details	Minor Allele	Frequency	References
*ALAD*	9q32	rs1800435	Exon 4	177 C>G (Lys59Asn)	G	1 and 3	[5,18]
*ATP7B*	13q14.3	rs1061472	Exon 10	2495 C>T (Lys832Arg)	T	27 and 48	[20,24]
*ATP7B*	13q14.3	rs732774	Exon 12	2855 T>C (Arg952Lys)	C	47	[20]
*CYP3A4*	7q22.1	rs2740574	Promoter	−392T>C	C	1–54 (range) ^a^	[22]
*CYP3A5*	7q22.1	rs776746	Exon 14	6986 C>T (Splice Defect)	T	6–55 (range) ^a^	[22]
*CYP3A7*	7q22.1	rs2257401	Exon 11	26041 G>C (Thr409Arg)	C	46–92 (range) ^a^	[22]
*FADS2*	11q12.2	rs174602	Intron	T>C (Asp6Asp)	C	18	[24]
*FADS2*	11q12.2	rs74771917	Intron	C>T	T	27	[24]
*FADS3*	11q12.2	rs7115739	Intron	1287-380 G>T	T	27	[24]
*GCLC*	6p12.1	rs17883901	Promoter	−129G>A	A	8 ^f^	[18,20,23]
*GCLM*	6p12.1	rs41303970	Promoter	−588 G>A	A	12 and 30	[15,23]
*GPX1*	3p21.3	rs1050450	Exon 2	559G>A (Pro198Leu)	A	3 and 25	[19,23]
*GPX4*	19p13.3	rs713041	3′UTR	718 C>T	T	11	[24]
*GSTP1*	11p13.2	rs1695	Exon 5	313 A>G (Ile105Val)	G	18–47 (range) ^b^	[15,18,20,23,25]
*MT1A*	16q13	rs8052394	Exon	152 A>G (Lys51Arg)	G	22	[26]
*MT1M*	16q13	rs2270836	Intron 2	95-49 C>T	T	22 and 35	[20,23]
*MT1M*	16q13	rs2270837	3′UTR	A>G	G	17–18 ^c^ (range)/24 ^d^	[16]
*MT1M*	16q13	rs9936741	Intron 2	31 T>C	C	13	[23]
*MT2A*	16q13	rs10636	3′UTR	+838 G>C	C	23–25 ^c^/23–25 ^d^ (range) ^e^	[16,20,23]
*MT4*	16q13	rs11643815	3′UTR	G>A Gly48Asp	A	0.6 and 9	[20,23]
*MTHFR*	1p36.22	rs2274976	Exon 11	1793 C>T (Arg594Gln)	T	9 and 49	[20,24]
*PON1*	7q21.3	rs705379	Exon 6	−108 G>A	A	37	[17]

Chr = Chromosome; UTR = untranslated region; ^a^ five cohorts were studied in this study: NC1-Seychelles (54.1, 54.5 and 44.8%), NC2—Seychelles (54.0, 55.1, and 46.5%), INMA—Spain (3.5, 7.8, and 90.5%), PHIME—Italy (3.2, 5.7, and 92.2%), and PHIME—Greece (1.4, 5.9 and 87.6; ^b^ Barcelos et al., 2013 [15] and 2015 [18] (43%), Chan et al., 2020 [23] (18%), Parajuli et al., 2016 [20] (33%), and Rahbar et al., 2021 [25] (47%). ^c^ Range of boys at entry (baseline) and in Year 2 and Year 7 of the study. ^d^ Range of girls at entry (baseline) and in Year 2 and Year 7 of the study; ^e^ Chan et al., 2020 [23] and Parajuli et al., 2016 [20] presented 22% and 25% of the variant allele, respectively; %). ^f^ Barcelos et al., 2015 [18] combined the heterozygous and variant homozygous genotypes for some SNPs, making it not possible to extract the variant allele frequency.

**Table 4:** Summary of the significant associations of the SNPs with the levels of mercury and the neurological symptoms.

Gene	Biological Pathways	SNP	Allele or Genotypes ^a^	Hg Levels	Neurological Outcomes	Reference
*ALAD*	Heme Biosynthesis	rs1800435	CG	High	-	[18]
*ATP7B*	Copper Transport	rs1061472	CT and TT	Low	-	[20]
*ATP7B*		rs732774	TC and CC	Low	-	[20]
*CYP3A4*	Xenobiotics Metabolism	rs2740574	TC + CC	High ^b^	Yes ^e^	[22]
*CYP3A5*		rs776746	CT + TT	High ^c^	Yes ^e^	[22]
*CYP3A7*		rs2257401	GC	High ^d^	Yes ^e^	[22]
*FADS2*	Fatty Acids Synthesis	rs174602	CC	Low	-	[24]
*FADS2*		rs74771917	TT	Low	-	[24]
*FADS3*		rs7115739	TT	Low	-	[24]
*GCLC*	Glutathione Synthesis	rs17883901	A	Low	-	[23]
*GCLM*		rs41303970	AA	Low	-	[15]
*GPX1*	Detoxification of hydrogen peroxide by glutathione peroxidase	rs1050450	A	Low	-	[23]
*GPX4*		rs713041	T and TT	Low	-	[24]
*GSTP1*	Glutathione	rs1695	G	High	-	[23]
*GSTP1*		rs1695	AG	High ^f^	-	[25]
*MT1A*	Metallothionein	rs8052394	AG + GG	High ^g^	Yes ^h^	[26]
*MT1M*		rs9936741	C	Low	-	[23]
*MT1M*		rs2270836	CT and TT	High	-	[20]
*MT1M*		rs2270837	AG	No	Yes ^i^	[16]
*MT2A*		rs10636	GC + CC	No	Yes ^i^	[16]
*MT4*		rs11643815	GA and AA	High	-	[20]
*MTHFR*	Metionina Biosynthesis	rs2274976	T and TT	Low	-	[24]
*PON1*	Lactonase and ester hydrolase activity	rs705379	GA and AA	Low	-	[17]

Yes—means that neurological outcomes were evaluated in association with the SNP. (-) The information was not evaluated in the article. ^a^ Minor alleles or variant genotypes (heterozygous and homozygous variant). ^b^ In one cohort (Spain), children carrying the *CYP3A4* TC and CC genotypes had elevated cord blood Hg concentrations. ^c^ In two cohorts (Spain and Greece), children carrying the *CYP3A5* CT and TT genotypes had elevated cord blood Hg concentrations. ^d^ In one cohort (Mediterranean), children carrying the *CYP3A7* GC genotype had elevated cord blood Hg concentrations. ^e^ Children had improved scores on the mental development index (MDI). ^f^ Mean blood Hg concentration in typically developing children compared to children with autism spectrum disorder status. ^g^ Individuals carrying *MT1A* AG and GG genotypes had both high blood Hg and an increased risk of cognitive impairment. ^h^ Individuals had significantly lower total scores (visuospatial/executive, attention, and memory domains). ^i^ Boys had significantly modified the adverse effects of Hg exposure on a number of neurobehavioral performance test outcomes, but this has little or no effect in similarly genotyped girls.

## Data Availability

Not applicable.

## Supplementary Materials

The following supporting information can be downloaded at: https://www.mdpi.com/article/10.3390/toxics12030226/s1, Table S1: Details of genes and SNPs studied in 13 included articles.

## References

[1] National Human Genome Research Institute. https://www.genome.gov/genetics-glossary.

[2] National Human Genome Research Institute Polymorphism. https://www.genome.gov/genetics-glossary/Polymorphism.

[3] Chiarella P., Capone P., Sisto R. (2023). Contribution of Genetic Polymorphisms in Human Health. Int. J. Environ. Res. Public Health.

[4] Basta P.C., Viana P.V.D.S., Vasconcellos A.C.S.D., Périssé A.R.S., Hofer C.B., Paiva N.S., Kempton J.W., Ciampi de Andrade D., Oliveira R.A.A.D., Achatz R.W. (2021). Mercury Exposure in Munduruku Indigenous Communities from Brazilian Amazon: Methodological Background and an Overview of the Principal Results. Int. J. Environ. Res. Public Health.

[5] Perini J.A., Silva M.C., Vasconcellos A.C.S.D., Viana P.V.S., Lima M.O., Jesus I.M., Kempton J.W., Oliveira R.A.A., Hacon S.S., Basta P.C. (2021). Genetic Polymorphism of Delta Aminolevulinic Acid Dehydratase (ALAD) Gene and Symptoms of Chronic Mercury Exposure in Munduruku Indigenous Children within the Brazilian Amazon. Int. J. Environ. Res. Public Health.

[6] Silva M.C., de Oliveira R.A.A., Vasconcellos A.C.S., Rebouças B.H., Pinto B.D., Lima M.O., de Jesus I.M., Machado D.E., Hacon S.S., Basta P.C. (2023). Chronic Mercury Exposure and GSTP1 Polymorphism in Munduruku Indigenous from Brazilian Amazon. Toxics.

[7] Feng X., Li P., Fu X., Wang X., Zhang H., Lin C.J. (2022). Mercury pollution in China: Implications on the implementation of the Minamata Convention. Environ. Sci. Process. Impacts.

[8] Oliveira R.A.A.D., Pinto B.D., Rebouças B.H., Ciampi de Andrade D., Vasconcellos A.C.S.D., Basta P.C. (2021). Neurological Impacts of Chronic Methylmercury Exposure in Munduruku Indigenous Adults: Somatosensory, Motor, and Cognitive Abnormalities. Int. J. Environ. Res. Public Health.

[9] Andreoli V., Sprovieri F. (2017). Genetic aspects of susceptibility to mercury toxicity: An overview. Int. J. Environ. Res. Public Health.

[10] National Library of Medicine National Center for Biotechnology Information. https://www.ncbi.nlm.nih.gov/snp/.

[11] SNPedia. https://www.snpedia.com/index.php.

[12] Von E.E. (2014). Strengthening the reporting of observational studies in epidemiology (STROBE) statement: Guidelines for reporting observational studies. Brit. Med. J..

[13] Cuschieri S. (2019). The CONSORT statement. Saudi J. Anaesth..

[14] Canva. https://www.canva.com/pt_br/.

[15] Barcelos G.R.M., Grotto D., de Marco K.C., Valentini J., Lengert A.H., de Oliveira A.A.S., Garcia S.C., Braga G.U.L., Engström K.S., Cólus I.M.D. (2013). Polymorphisms in glutathione-related genes modify mercury concentrations and antioxidant status in subjects environmentally exposed to methylmercury. Sci. Total Environ..

[16] Woods J.S., Heyer N.J., Ruesso J.E., Martin M.D., Pillai P.B., Farin F.M. (2013). Modification of neurobehavioral effects of mercury by genetic polymorphisms of metallothionein in children. Neurotoxicol. Teratol..

[17] Drescher O., Dewailly E., Diorio C., Ouellet N., Sidi E.A.L., Abdous B., Valera B., Ayotte P. (2014). Methylmercury exposure, PON1 gene variants and serum paraoxonase activity in Eastern James Bay Cree adults. J. Expo. Sci. Environ. Epidemiol..

[18] Barcelos G.R.M., de Souza M.F., de Oliveira A.A.S., Lengert A.H., de Oliveira M.T., Camargo R.B.O.G., Grotto D., Valentini J., Garcia S.C., Braga G.U.L. (2015). Effects of genetic polymorphisms on antioxidant status and concentrations of the metals in the blood of riverside Amazonian communities co-exposed to Hg and Pb. Environ. Res..

[19] Rocha A.V., Cardoso B.R., Zavarize B., Almondes K., Bordon I., Hare D., Favaro B.I.T., Cozzolino S.M.F. (2016). GPX1 Pro198Leu polymorphism and GSTM1 deletion do not affect selenium and mercury status in mildly exposed Amazonian women in an urban population. Sci. Total Environ..

[20] Parajuli R.P., Goodrich J.M., Chou H.-N., Gruninger S.E., Dolinoy D.C., Franzblau A., Basu N. (2016). Genetic Polymorphisms Are Associated with Hair, Blood, and Urine Mercury Levels in the American Dental Association (ADA) Study Participants. Environ. Res..

[21] Lopes A.C.B.A., Urbano M.R., Souza-Nogueira A., Oliveira-Paula G.H., Michelin A.P., Carvalho M.F.H., Camargo A.E.I., Peixe T.S., Cabrera M.A.S., Paoliello M.M.B. (2017). Association of lead, cadmium and mercury with paraoxonase 1 activity and malondialdehyde in a general population in Southern Brazil. Environ. Res..

[22] Llop S., Tran V., Ballester F., Sofianou-Katsoulis A., Sunyer J., Engström K., Alhamadow A., Love T.M., Watson G.E., Bustamante M. (2017). CYP3A genes and the association between prenatal methylmercury exposure and neurodevelopment. Environ. Int..

[23] Chan P.H.Y., Chan K.Y.Y., Schooling C.M., Hui L.L., Chan M.H.M., Li A.M., Cheung R.C.K., Lam H.S. (2020). Association between Genetic Variations in GSH-Related and MT Genes and Low-Dose Methylmercury Exposure in Children and Women of Childbearing Age: A Pilot Study. Environ. Res..

[24] Parajuli R.P., Goodrich J.M., Chan H.M., Lemire M., Ayotte P., Hegele R.A., Basu N. (2021). Variation in Biomarker Levels of Metals, Persistent Organic Pollutants, and Omega-3 Fatty Acids in Association with Genetic Polymorphisms among Inuit in Nunavik, Canada. Environ. Res..

[25] Rahbar M.H., Samms-Vaughan M., Saroukhani S., Bressler J., Hessabi M., Grove M.L., Shakspeare-Pellington S., Loveland K.A., Beecher C., Mclaughlin W. (2021). Associations of Metabolic Genes (GSTT1, GSTP1, GSTM1) and Blood Mercury Concentrations Differ in Jamaican Children with and without Autism Spectrum Disorder. Int. J. Environ. Res. Public Health.

[26] Sirivarasai J., Chaisungnern K., Panpunuan P., Chanprasertyothin S., Chansirikanjana S., Sritara P. (2021). Role of MT1A Polymorphism and Environmental Mercury Exposure on the Montreal Cognitive Assessment (MoCA). Neuropsychiatr. Dis. Treat..

[27] FAO Joint, WHO Expert Committee on Food Additives, World Health Organization (1989). Evaluation of certain food additives and contaminants (Thirty-third report of the Joint FAO/WHO Expert Committee on Food Additives). WHO Technical Report Series.

[28] Legrand M., Feeley M., Tikhonov C., Schoen D., Li-Muller A. (2010). Methylmercury blood guidance values for Canada. Can. J. Public Health.

[29] WHO (World Health Organization) (2008). Guidance for Identifying Populations at Risk from Mercury Exposure.

[30] Llop S., Balallester F., Broberg K. (2015). Effect of gene-mercury interactions on mercury toxicokinetics and neurotoxicity. Curr. Environ. Health Rep..

[31] Manikandan P., Nagini S. (2017). Cytochrome P450 structure, function and clinical significance: A review. Curr. Drug Targets.

[32] Klyushova L.S., Perepechaeva M.L., Grishanova A.Y. (2022). The role of CYP3A in health and disease. Biomedicines.

[33] Lamba J.K., Lin Y.S., Schuetz E.G. (2002). Thummel KE. Genetic contribution to variable human CYP3A-mediated metabolism. Adv. Drug Deliv. Rev..

[34] Rodríguez-Antona C., Jande M., Rane A., Ingelman-Sundberg M. (2005). Identification and phenotype characterization of two CYP3A haplotypes causing different enzymatic capacity in fetal livers. Clin. Pharmacol. Ther..

[35] Suarez-Kurtz G., Perini J.A., Bastos-Rodrigues L., Pena S.D., Struchiner C. (2007). Impact of population admixture on the distribution of the CYP3A5*3 polymorphism. Pharmacogenomics.

[36] Sim S.C., Ingelman-Sundberg M. (2010). The Human Cytochrome P450 (CYP) Allele Nomenclature website: A peer-reviewed database of CYP variants and their associated effects. Hum. Genom..

[37] Chernyak Y.I., Merinova A.P. (2020). CYP3A Polymorphism and Chronic Mercury Intoxication. Bull. Exp. Biol. Med..

[38] Smith C.M., Wang X., Hu H., Kelsey K.T. (1995). A polymorphism in the delta-aminolevulinic aciddehydratase gene may modify thepharmacokinetics and toxicity of lead. Environ. Health Perspect..

[39] Ballatori N., Clarkson W. (1985). Biliary Secretion of Glutathione and of Glutathione-Metal Complexes. Fundam. Appl. Toxicol..

[40] Gundacker C., Wittmann K.J., Kukuckova M., Komarnicki G., Hikkel I., Gencik M. (2009). Genetic Background of Lead and Mercury Metabolism in a Group of Medical Students in Austria. Environ. Res..

[41] Wahlberg K., Love T.M., Pineda D., Engström K., Watson G.E., Thurston S.W., Yeates A.J., Mulhern M.S., McSorley E.M., Strain J.J. (2018). Maternal Polymorphisms in Glutathione-Related Genes Are Associated with Maternal Mercury Concentrations and Early Child Neurodevelopment in a Population with a Fish-Rich Diet. Environ. Int..

[42] Medina Pérez O.M., Flórez-Vargas O., Rincón Cruz G., Rondón González F., Rocha Muñoz L., Sánchez Rodríguez L.H. (2021). Glutathione-Related Genetic Polymorphisms Are Associated with Mercury Retention and Nephrotoxicity in Gold-Mining Settings of a Colombian Population. Sci. Rep..

[43] Lozano M., Murcia M., Soler-Blasco R., González L., Iriarte G., Rebagliato M., Lopez-Espinosa M.J., Esplugues A., Ballester F., Llop S. (2021). Exposure to mercury among 9-year-old children and neurobehavioural function. Environ. Int..

[44] Irvine G.W., Pinter T.B., Stillman M.J. (2016). Defining the metal binding pathways of human metallothionein 1A: Balancing zinc availability and cadmium seclusion. Metallomics.

[45] Babula P., Masarik M., Adam V., Eckschlager T., Stiborova M., Trnkova L., Skutkova H., Provaznik I., Hubalek J., Kizek R. (2012). Mammalian metallothioneins: Properties and functions. Metallomics.

[46] Wang Y., Goodrich J.M., Gillespie B., Werner R., Basu N., Franzblau A. (2012). An investigation of modifying effects of metallothionein single-nucleotide polymorphisms on the association between mercury exposure and biomarker levels. Environ. Health Perspect..

[47] Engstrom K.S., Stromberg U., Lundh T., Johannsson I., Vessby B., Hallmans G., Skerfving S., Broberg K. (2008). Genetic variation in glutathione-related genes and body burden of methylmercury. Environ. Health Perspect..

[48] Gundacker C., Komarnicki G., Jagiello P., Gencikova A., Dahmen N., Wittmann K.J., Gencik M. (2007). Glutathione-S-transferase polymorphism, metallothionein expression, and mercury levels among students in Austria. Sci. Total Environ..

[49] Goodrich J.M., Chou H.N., Gruninger S.E., Franzblau A., Basu N. (2016). Exposures of dental professionals to elemental mercury and methylmercury. J. Expo. Sci. Environ. Epidemiol..

[50] Romo L., Findlay S.D., Burge C.B. (2024). Regulatory features aid interpretation of 3′UTR variants. Am. J. Hum. Genet..

[51] Moleirinho A., Carneiro J., Matthiesen R., Silva R.M., Amorim A., Azevedo L. (2011). Gains, losses and changes of function after gene duplication: Study of the metallothionein family. PLoS ONE.

[52] Antunes Dos Santos A., Appel Hort M., Culbreth M., López-Granero C., Farina M., Rocha J.B., Aschner M. (2016). Methylmercury and brain development: A review of recent literature. J. Trace Elem. Med. Biol..

[53] Grandjean P., Weihe P., White R.F., Debes F., Araki S., Yokoyama K., Murata K., Sørensen N., Dahl R., Jørgensen P.J. (1997). Cognitive deficit in 7-year-old children with prenatal exposure to methylmercury. Neurotoxicol. Teratol..

[54] Debes F., Budtz-Jørgensen E., Weihe P., White R.F., Grandjean P. (2006). Impact of prenatal methylmercury exposure on neurobehavioral function at age 14 years. Neurotoxicol. Teratol..

