# Supplementary material for: Single-Nucleotide Polymorphisms Associated with Mercury Levels and Neurological Symptoms: An Overview
Source: Toxics. 2024 Mar 20;12(3):226. doi: 10.3390/toxics12030226 (PMC10975854; doi:10.3390/toxics12030226)
Supplement: Supplementary file 1 [file toxics-12-00226-s001.zip › toxics-2905208-supplementary.pdf]

Table S1. Details of genes and SNPs studied in 13 included articles.

Gene	Chr	dnSNP ID	Gene locus	SNP details	Genotyping method	Minor allele	Frequency	References
ABCB1	7q21.12	rs1045642	Exon 26	3435 A>G (Ile1145Ile)	MassArray	G	48	[20]
ABCB1	7q21.12	rs17064	3'UTR	89 T>A	MassArray	A	6	[20]
ABCB1	7q21.12	rs3842	3'UTR	202 T>C	MassArray	C	2	[20]
ABCB1	7q21.12	rs9282564	Exon 2	61 T>C (Asn21Asp)	MassArray	C	6	[20]
ABCC1	16p13.11	rs212090	3'UTR	543 T>A	MassArray	A	20	[24]
ABCC1	16p13.11	rs2230671	Exon 28	4002 G>A (Ser1334Ser)	MassArray	A	24	[20]
ABCC1	16p13.11	rs8187858	Exon 13	1704 C>T (Tyr568Tyr)	MassArray	T	8	[20]
ABCC2	10q24.2	rs17222723	Exon 25	3563 T>A (Val1188Glu)	MassArray	A	5	[20]
ABCC2	10q24.2	rs1885301	5'UTR	-1549 A>G	MassArray	G	15	[24]
ABCC2	10q24.2	rs2273697	Exon 10	1249 G>A (Val417Ile)	MassArray	A	17	[20]
ABCC2	10q24.2	rs3740066	Exon 28	3972 C>T (Ile1324Ile)	MassArray	T	15	[24]
ABCC2	10q24.2	rs8187710	Exon 32	4544G>A (Cys1515Tyr)	MassArray	A	5	[20]
ABCC4	13q32.1	rs868853	Promoter	−1508 T>C	MassArray	C	1	[20]
ABCG2	4q22.1	rs2231142	Exon 5	421 G>T (Glu141Lys)	MassArray	T	15	[20]
AHCY	20q11.22	rs819146	5'UTR	-124 T>G	MassArray	G	15	[20]
ALAD	9q32	rs1800435	Exon 4	177 C>G (Lys59Asn)	TaqMan	G	1 and 3	[5,18]
ATP7B	13q14.3	rs1061472	Exon 10	2495 C>T (Lys832Arg)	MassArray	T	27 and 48	[20,24]
ATP7B	13q14.3	rs1801243	Exon 2	1216 A>C (Ser406Ala)	MassArray	C	27 and 48	[20,24]
ATP7B	13q14.3	rs732774	Exon 12	2855 T>C (Arg952Lys)	MassArray	C	47	[20]
BCL11A[a]	2p16.1	rs4671393	Intron 2	G>A	MassArray	A	2	[20]
BDNF[a]	11p14.1	rs6265	Exon 11	196 C>T (Val66Met)	MassArray	T	29	[20]
CAT[a]	11p13	rs7943316	Promoter	−21 A>T	MassArray	T	10 and 43	[24,20]
CBS	21q22.3	rs1051319	3'UTR	123 C>G	MassArray	G	7	[20]
CBS	21q22.3	rs2850146	Promoter	-8283 C>G	MassArray	G	8	[20]
CPOX	3q11.2	rs1131857	Exon 4	814 T>G (Asn272His)	MassArray	G	18	[20]
CYP2D6	22q13.2	rs1080985	Promoter	-1584 G>C	MassArray	C	27	[24]
CYP3A4	7q22.1	rs2740574	Promoter	−392T>C	TaqMan and Microarray[b]	C	1-54 (range)[f]	[22]
CYP3A5	7q22.1	rs776746	Exon 14	6986 C>T (Splice Defect)	TaqMan and Microarray[b]	T	6-55 (range)[f]	[22]
CYP3A7	7q22.1	rs2257401	Exon 11	26041 G>C (Thr409Arg)	TaqMan	C	46-92 (range)[f]	[22]
DNMT1	19p13.2	rs2228613	Exon	1632 G>T (Ile544Ile)	MassArray	T	6	[20]
FADS2	11q12.2	rs174602	Intron	T>C (Asp6Asp)	MassArray	C	18	[24]
FADS2	11q12.2	rs74771917	Intron	C>T	MassArray	T	27	[24]
FADS3	11q12.2	rs7115739	Intron	1287-380 G>T	MassArray	T	27	[24]
GCLC	6p12.1	rs17883901	Promoter	-129G>A	TaqMan and MassArray[c]	A	8[g]	[18,23,20]

GCLM	6p12.1	rs41303970	Promoter	-588 G>A	TaqMan	A	12 and 30	[15,23]
GGTLC1	20p11.21	rs395485	Exon 8	C>T	MassArray	T	32	[20]
GLRX2[a]	1q31.2	rs912071	5'UTR	T>C	MassArray	C	37	[20]
GPX1	3p21.3	rs1050450	Exon 2	559G>A (Pro198Leu)	TaqMan	A	3 and 25	[23,19]
GPX1	3p21.3	rs1800668	5'UTR	+2G>A	TaqMan	A	-	[18]
GPX4	19p13.3	rs713041	3'UTR	718 C>T	MassArray	T	11	[24]
GPX6	6p22.1	rs6413428	Exon 5	710 A>G	MassArray	G	18	[20]
GPX7	1p32.3	rs1970951	Exon 2	237 C>T (Phe79Phe)	MassArray	T	17	[20]
GSS	20q11.22	rs3761144	5'UTR	-4420G>C	MassArray	C	42	[20]
GSS	20q11.22	rs725521	3'UTR	559C>T	MassArray	T	42	[20]
GSTA2	6p12.2	rs2180314	Exon 5	63G>C (Ser112Thr)	MassArray	C	4	[20]
GSTA4	6p12.2	rs367836	3'UTR	+137 T>G	MassArray	G	34	[20]
GSTA4	6p12.2	rs405729	3'UTR	668 C>T	MassArray	T	35 and 39	[24,20]
GSTM1	1p13.3	rs1065411	Exon 7	2697 G>C (Lys173Asn)	MassArray	C	17	[20]
GSTM3	1p13.3	rs1332018	Promoter	-63 T>G	MassArray	G	34	[20]
GSTM3	1p13.3	rs7483	Exon 7	C>T (Val224Ile)	MassArray	T	43	[20]
GSTO1	10q25.1	rs4925	Exon 4	419 C>A(Ala140Asp)	MassArray	A	27	[20]
GSTO2	10q25.1	rs156697	Exon 5	424 A>G (Asn142Asp)	MassArray	G	36	[20]
GSTP1	11p13.2	rs1138272	Exon 6	341 C > T (Ala114Val)	MassArray	T	6	[20]
GSTP1	11p13.2	rs1695	Exon 5	313 A>G (Ile105Val)	TaqMan and MassArray[c]	G	18-47 (range)[h]	[15,18,23,20, 25]
HBS1L	6q23.3	rs4895441	Intergenic	A>G	MassArray	G	26	[20]
HMOX1[a]	22q12.3	rs2071747	Exon 1	19 G>C (Asp7His)	MassArray	C	5	[20]
MDR1	38.p14	rs2032582	Exon 1	2677 C>A (Ala893Ser/Thr)	TaqMan	A	47	[18]
MT1A	16q13	rs11076161	Intron 1	G>A	MassArray	A	32	[20]
MT1A	16q13	rs8052394	Exon	152 A>G (Lys51Arg)	PCR-RFLP	G	22	[26]
MT1B	16q13	rs7191779	Exon	-1975 C>G	MassArray	G	48	[20]
MT1B	16q13	rs8052334	Promoter	95-68 T>C	MassArray	C	48	[20]
MT1B	16q13	rs964372	Intron 1	G>C	MassArray	C	18	[20]
MT1E	38.p14	rs7403881	5'UTR	G>C	MassArray	C	46	[20]
MT1M	16q13	rs2270836	Intron 2	95-49 C>T	TaqMan and MassArray[c]	T	22 and 35	[23,20]
MT1M	16q13	rs2270837	3'UTR	A>G	TaqMan	G	17-18[i] (range) / 24[j]	[16]
MT1M	16q13	rs9936741	Intron 2	31 T>C	TaqMan	C	13	[23]
MT2A	16q13	rs10636	3'UTR	+838 G>C	TaqMan and MassArray[c]	C	23-25[i] / 23-25[j] (range)[k]	[23,20,16]
MT3	16q13	rs11644094	Intron	A>G	MassArray	G	32	[20]
MT4	16q13	rs11643815	3'UTR	G>A Gly48Asp	TaqMan and MassArray[c]	A	0.6 and 9	[23,20]
MTF1	1p34.3	rs12751325	Intron	T>C	MassArray	C	28	[20]

MTF1	1p34.3	rs473279	Exon	C>T	MassArray	T	13	[24]
MTF1	1p34.3	rs9660548	5'UTR	8298155 T>A	MassArray	A	42	[24]
MTHFR	1p36.22	rs1801133	Exon 11	677 G>A (Ala222Val)	MassArray	A	14	[24]
MTHFR	1p36.22	rs2274976	Exon 11	1793 C>T (Arg594Gln)	MassArray	T	9 and 49	[20,24]
MTR	1q43	rs1805087	Exon 26	2756 A>G (Asp919Gly)	MassArray	G	15	[20]
MTRR	1p36.22	rs1801131	Exon 4	1298 T>G (Gln429Ala)	MassArray	G	46	[24]
MTRR	5p15.31	rs1801394	Exon 7	66 G>A (Ile22Met)	MassArray	A	27 and 47	[24,20]
MTRR	5p15.31	rs3776467	Intron	7068 A>G	MassArray	G	37	[20]
NOS1[a]	12q24.22	rs2682826	Exon 2	151745 G>A	MassArray	A	27	[20]
PON1	7q21.3	rs662	3'UTR	192 T>C (Gln192Arg)	Spectrophotometer[d] and MassArray or TaqMan[e]	C	37 and 56	[21,17]
PON1	7q21.3	rs705379	Exon 6	-108 G>A	MassArray or TaqMan[e]	A	37	[17]
PON1	7q21.3	rs705381	Promoter	-162 C>T	MassArray or TaqMan[e]	T	12	[17]
PON1	7q21.3	rs854560	Promoter	163 T>A (Leu54Met)	MassArray or TaqMan[e]	A	18	[17]
PON1	7q21.3	rs854571	Promoter	−832 C>T	MassArray or TaqMan[e]	T	41	[17]
PON1	7q21.3	rs854572	Exon 3	−909 C>G	MassArray or TaqMan[e]	G	39	[17]
PRDX2[a]	19p13.13	rs10427027	3′UTR	T>C	MassArray	C	11	[20]
PRDX2[a]	19p13.13	rs12151144	Intron	A>C	MassArray	C	11	[20]
PRDX6[a]	1q25.1	rs33942654	Promoter	G>A	MassArray	A	23	[20]
SCL22A8	11q12.3	rs4149182	Intron	437+79C>G	MassArray	G	23	[20]
SELS	15q26.3	rs7178239	5'UTR	C>G	MassArray	G	22	[20]
SEPHS2	16p11.2	rs1133238	3'UTR	G>A	MassArray	A	8	[20]
SEPN1	1p36.11	rs2294228	Exon 4	1506A>C (Asn502Lys)	MassArray	C	17	[20]
SEPN1	1p36.11	rs7349185	3'UTR	425A>G (Tyr142Cys)	MassArray	G	15	[20]
SEPP1	1p36.11	rs3877899	Exon 5	700 C>T (Ala234Ser)	MassArray	T	18	[20]
SEPP1	5p12	rs7579	Exon	25191 C>T	MassArray	T	28	[20]
SLC11A1	2q25	rs1059823	3′UTR	577 A>G	MassArray	G	38	[20]
SLC11A1	2q25	rs3731865	Intron 4	469+14G>C	MassArray	C	22	[20]
SLC22A6	11q12.3	rs4149170	3'UTR	453 C>T	MassArray	T	11	[20]
SLC22A8	11q12.3	rs4149182	Intron	C>G	MassArray	G	23	[20]
SLC3A2	11q12.3	rs2269353	5'UTR	G>A	MassArray	A	8	[20]
SLC43A2	17p13.3	rs4790732	5'UTR	G>C	MassArray	C	34 and 49	[20,24]
SLC7A2	8p22	rs62622371	Exon	G>A	MassArray	A	7	[20]
SLC7A5	16q24.2	rs33916661	5'UTR	A>G	MassArray	G	2	[20]
SLC7A7	14q11.2	rs2281677	Intron	-86 G>A	MassArray	A	37	[20]
SLC7A8	14q11.2	rs8011016	Intron	T>C	MassArray	C	14	[20]
SLCO1B1	12p12.1	rs2306283	Intron	388 A>G (Asn130Asp)	MassArray	G	34	[24]

TET1	10q21.3	rs10823229	Exon	A>G (Asp162Gly)	MassArray	G	39	[20]
TET1	10q21.3	rs3998860	Exon	3369 G>A (Ile1123Met)	MassArray	A	16	[20]
TET2	4q24	rs34402524	Exon 11	5162T>G (Leu1721Trp)	MassArray	G	11	[20]
TXNRD2[a]	22q11.21	rs5748469	Exon 4	196 C>A (Ala66Ser)	MassArray	A	49	[20]
TXNRD2[a]	22q11.21	rs5992495	Exon	865 T>G (Ser299Arg)	MassArray	G	16	[20]
TXNRD3[a]	3q21.3	rs3108755	Exon 3	G>A	MassArray	A	5	[20]
VDR[a]	12q13.11	rs1544410	5'UTR	63980 C>T (BsmI)	TaqMan	T	32	[18]

(-) no information; Chr = Chromosome; UTR = untranslated region; MassArray = Sequenom iPLEX Gold platform; TaqMan = Real-Time PCR; [a]Genes in toxicodynamic pathway of mercury; [b]Genotyping of rs776746 and rs2740574 in INMA cohort was performed by Microarray technology (Illumine Human Omni1-Quad BeadChip platform); [c]Parajuli et al.,[20] 2016 used MassArray technology; [d]Lopes et al., [21] 2017 used EnSpire spectrophotometer microplate reader platform; [e]The authors did not discriminate which technology was used for each SNP. [f]Five cohorts were studied in this study: NC1-Seychelles (54.1, 54.5 and 44.8%), NC2-Seychelles (54.0, 55.1 and 46.5%), INMA-Spain (3.5, 7.8 and 90.5%), PHIME-Italy (3.2, 5.7 and 92.2%) and PHIME-Greece (1.4, 5.9 and 87.6%). [g]Barcelos et al., 2015 combined the heterozygous and variant homozygous genotypes for some SNPs, making it not possible to extract the variant allele frequency. [h]Barcelos et al., 2013 [15] and 2015 (43%) [18] , Chan et al., [23]((18%), Parajuli 2016 [20] (et al., (33%), and Rahbar et al., [25] (47%). [i]Range of boys at Entry (Baseline) and in Year 2 and Year 7 of study. [j]Range of girls at Entry (Baseline) and in Year 2 and Year 7 of study; [k]Chan et al., and Parajuli et al., 2016 presented 22% and 25% of the variant allele, respectively.
